# Specific Pyruvate Kinase M2 Inhibitor, Compound 3K, Induces Autophagic Cell Death through Disruption of the Glycolysis Pathway in Ovarian Cancer Cells

**DOI:** 10.7150/ijbs.59855

**Published:** 2021-05-05

**Authors:** Jae Hyeon Park, Amit Kundu, Su Hyun Lee, ChunXue Jiang, Song Hee Lee, Ye Seul Kim, So Young Kyung, So Hyun Park, Hyung Sik Kim

**Affiliations:** School of Pharmacy, Sungkyunkwan University, Suwon 16419, Republic of Korea.

**Keywords:** compound 3K, ovarian cancer, pyruvate kinase M2, autophagy, apoptosis

## Abstract

Ovarian cancer is a common cause of death among gynecological cancers. Although ovarian cancer initially responds to chemotherapy, frequent recurrence in patients remains a therapeutic challenge. Pyruvate kinase M2 (PKM2) plays a pivotal role in regulating cancer cell survival. However, its therapeutic role remains unclear. Here, we investigated the anticancer effects of compound 3K, a specific PKM2 inhibitor, on the regulation of autophagic and apoptotic pathways in SK-OV-3 (PKM2-overexpressing human ovarian adenocarcinoma cell line). The anticancer effect of compound 3K was examined using MTT and colony formation assays in SK-OV-3 cells. PKM2 expression was positively correlated with the severity of the tumor, and expression of pro-apoptotic proteins increased in SK-OV-3 cells following compound 3K treatment. Compound 3K induced AMPK activation, which was accompanied by mTOR inhibition. Additionally, this compound inhibited glycolysis, resulting in reduced proliferation of SK-OV-3 cells. Compound 3K treatment suppressed tumor progression in an *in vivo* xenograft model. Our findings suggest that the inhibition of PKM2 by compound 3K affected the Warburg effect and induced autophagic cell death. Therefore, use of specific PKM2 inhibitors to block the glycolytic pathway and target cancer cell metabolism represents a promising therapeutic approach for treating PKM2-overexpressing ovarian cancer.

## Introduction

Ovarian cancer is the most common gynecologic tumor of the female reproductive tract. In 2019, approximately 22,530 new cases of ovarian cancer were reported in the United States. Among females, ovarian cancer represents 5% of the total number of cancer-related deaths in the United States 1. Due to the lack of specific symptoms, ovarian cancer is often diagnosed at the metastatic stage, contributing to poor prognosis. Although most patients with ovarian cancer initially respond to standard therapies based on a combination of platinum-based chemotherapy and surgery, many patients develop resistance to chemotherapy and present with recurrent tumors. Therefore, the relative 10-year survival rate of ovarian cancer is 8% for stage IV 2.

In general, cancer cells use a high amount of glucose and secrete a large amount of lactate in the presence of abundant oxygen, in a phenomenon referred to as the “Warburg effect.” Glycolytic intermediates produced through the Warburg effect promote the biosynthesis of ATP and cellular macromolecular building blocks 3. Pyruvate kinase M2 (PKM2), the rate-limiting enzyme in the terminal step of glycolysis, plays one of the most important roles in the aerobic glycolysis pathway, which mediates the conversion of phosphoenolpyruvate to pyruvate to release energy, and thus, it provides favorable conditions for the growth of cancer cells 4.

Besides metabolic reprograming, PKM2 acts as a protein kinase transcriptional coactivator of genes that influence cell proliferation, apoptosis, and migration 5, 6. In the cellular environment, PKM2 is mechanistically associated with the mammalian target of rapamycin (mTOR)/protein kinase B (Akt) signaling pathway, which is associated with various cellular processes, including cell survival and growth, protein synthesis, cell cycle progression, and angiogenesis 7.

A previous study demonstrated that the overexpression of PKM2 activates mTORC1 by phosphorylating its substrate, AKT1 substrate 1, which in turn accelerates autophagy inhibition and oncogenic growth in cancer cells, leading to poor patient outcome 8. It has been demonstrated that autophagy is impaired by the activation of the Akt/mTOR pathway in ovarian cancer cells. Therefore, autophagy is heavily regulated by the kinase mTOR 9.

It has been established that the downregulation of PKM2 reduces cancer growth and induces cell death 10. The inhibition of PKM2 affects cell survival and proliferation in many types of cancer cells. Peptide aptamers and RNA interference-mediated ablation of PKM2 exerted an anticancer effect through the induction of the apoptosis pathway and sensitization to chemotherapy 11-13. A recent study reported the anticancer effect of small interfering pyruvate kinase M2 (siPKM2) against SK-OV-3 cell lines in which PKM2 is notably expressed and demonstrated that siPKM2 significantly reduced cell proliferation and induced apoptosis 14. However, specific PKM2 inhibitors, which can easily be used in clinical settings, have not been investigated in ovarian cancer.

Several PKM2 inhibitors, such as shikonin, metformin, vitamin K, and temozolomide have been reported 9. Recently, the biological effects of novel naphthoquinone derivatives as selective inhibitors of PKM2 were reported. Some compounds, such as compound 3K, exhibited more potent PKM2 inhibition than shikonin, the conventional PKM2 inhibitor. Compound 3K also showed significant antiproliferative activity in various cancer cell lines. The higher selectivity of compound 3K (IC_50_ [PKM1]/IC_50_ [PKM2] = 5.7) for PKM2 compared with that of the conventional PKM2 inhibitor, shikonin (IC_50_ [PKM1]/IC_50_ [PKM2] = 1.5), was validated using cell-free fluorescent lactate dehydrogenase (LDH) and pyruvate kinase (PK) coupled assay 15.

Several studies have explored the tumorigenic role of PKM2 toward the development of potential treatment strategies for various cancers 11-14, 16, 17. However, the therapeutic efficacy of the specific PKM2 inhibitor, compound 3K, in ovarian cancer remains unclear. In this study, we identified a relationship between PKM2 overexpression and ovarian cancer progression. We evaluated the anticancer effects of compound 3K in PKM2-overexpressing SK-OV-3 cells, a well-established human epithelial adenocarcinoma cell line model that is resistant to several cytotoxic drugs such as cis-platinum, diphtheria toxin, and adriamycin 18. The inhibition of PKM2 perturbed the glycolytic pathway and activated autophagic cell death by regulating the Akt/AMPK/mTOR pathway. These results suggest that the inhibition of PKM2 can enhance the inhibition of ovarian cancer cell growth by suppressing aerobic glycolysis, which provides a basis for the development of PKM2-targeted therapies for ovarian cancer through PKM2 overexpression.

## Materials and Methods

### Reagents

Compound 3K was obtained from SelleckChem (Houston, TX, USA). A solution of compound 3K (15 mM) was prepared in DMSO. Culture medium and fetal bovine serum (FBS) were obtained from Gibco Invitrogen Corporation (Carlsbad, CA, USA). Horseradish peroxidase (HRP)-conjugated secondary antibodies were obtained from Santa Cruz Biotechnology (Santa Cruz, CA, USA). Primary antibodies against PKM2, PKM1, Cyclin B1, p-Cdc2, Cdc2, Bax, Bcl-2, caspase 3, Beclin-1, LC3B, Atg5, Atg7, p62, MMP-2, MMP-9, TIMP-2, p-AKT, AKT, p-AMPK, AMPK, p-mTOR, mTOR, p-p70S6K, p70S6K, GLUT1, LDHA, MCT4, and β-actin were all obtained from Cell Signaling (Beverly, MA, USA). MTT and DAPI were obtained from Thermo Fisher Scientific (Invitrogen, NY, USA).

### Cell lines and cell culture

Human ovarian cancer cell lines (SK-OV-3, CA-OV-3, OVCAR3, and TOV-21G) were purchased from the American Type Culture Collection (Manassas, VA, USA). SK-OV-3, CA-OV-3, OVCAR3, and TOV-21G cells were grown in Roswell Park Memorial Institute (RPMI) 1640 supplemented with 10% FBS, 100 U/ml penicillin, and 100 μg/ml streptomycin (WelGENE, Daegu, South Korea) in a humidified atmosphere of 5% CO_2_ at 37 °C.

### Ovarian tissue microarray and immunohistochemistry (IHC)

Tissue microarray (consisting of 18 normal human ovarian and 143 ovarian adenocarcinoma samples) was purchased from US Biomax Inc. (Rockville, MD, USA) (Table 1). IHC analysis was performed to investigate the expression of PKM2 in the normal ovarian and ovarian adenocarcinoma tissue samples. The tissue microarray was treated with xylene and graded ethanol, followed by boiling in sodium citrate buffer for 15 min. The slide was treated with 5% H_2_O_2_ for 15 min, and then with PKM2 antibody (Cat. no. 4053, dilution, 1:1,000; Cell Signaling Technology, Inc., Danvers, MA, USA) for 24 h at 4 °C. The slides were treated with goat anti-rabbit IgG secondary antibody (VECTOR laboratories, Burlingame, CA, USA) for 30 min, followed by treatment with the HRP-streptavidin reagent (VECTOR laboratories) for 30 min. After staining with DAB (Dako, Agilent, Santa Clara, CA, USA) and subsequently with hematoxylin (Dako), the slides were fixed in the mounting solution after treatment with graded ethanol and xylene. The slides were viewed using a K1-fluo microscope (Nanoscope Systems, Daejeon, Korea) at 200× magnification. For the histological review, all tissue samples were evaluated by two pathologists. The average immunostaining intensity of PKM2 was determined using a four-category scale: 0 for negative staining, 1 for weak intensity, 2 for moderate intensity, and 3 for strong intensity. The percentage of cells with positive PKM2 immunostaining was calculated (range 0-100). The score for the proportion of stained cells and that for the corresponding intensity of staining were added to obtain the total PKM2 immunostaining score (range 0-300).

### Cytotoxicity assay

The cytotoxic effect of compound 3K was examined using MTT (1 mg/ml, Sigma-Aldrich) assay. The cells were seeded onto a 96-well plate at a density of 3×10^3^ cells/well for 24 h, followed by treatment with varying drug concentrations (1-15 µM) for 24 and 48 h. Next, 100 µl of MTT reagent was added at the endpoints, following which the cells were further incubated at 37 °C in the dark for 3 h. The absorbance of each well was measured at 540 nm using a VERSA Max Microplate Reader (Molecular Devices Corp., CA, and USA). The IC_50_ value was calculated using the Sigma Plot 10.0 software from sigmoidal dose-response relationships.

### Clonogenic assay

SK-OV-3 cells were grown in 6-well plates at a density of 1×10^3^ cells/well for 24 h, and the cells were subsequently treated with compound 3K (1, 2.5, or 5 µM) for 10 days. The cells were stained with 0.05% crystal violet for 3 h following the fixing of the colonies with 4% paraformaldehyde. Relative colony area was analyzed using an image analyzer.

### Western blot analysis

SK-OV-3 cells were harvested after 24 h of treatment with compound 3K (1, 2.5, or 5 µM). The cells were lysed and total protein was extracted with the PRO-PREP^TM^ extraction solution. Equal amounts of protein were separated on sodium dodecyl sulfate polyacrylamide gels and transferred onto a polyvinylidene difluoride (PVDF) membrane. The PVDF membranes were incubated with the primary antibodies overnight at 4 °C, washed with tris-buffered saline, followed by treatment with HRP-conjugated anti-mouse or anti-rabbit antibodies. The protein bands were visualized using a ChemiDoc imaging system (Bio-Rad, Hercules, CA, USA). The intensities of the bands were calculated using an image analyzer.

### AnnexinV-FITC binding assay

The Annexin V-FITC analysis was performed using the Annexin V-FITC staining kit I (BD Biosciences, San Diego, CA, USA). SK-OV-3 cells were treated with compound 3K (1, 2.5, or 5 µM) for 24 h. After the cells were harvested, 5 µl of Annexin V-FITC and propidium iodide (PI) each in 100 µl of binding buffer were added, followed by incubation for 15 min in the dark. After the addition of 400 µl of 1× binding buffer, the cells were analyzed using flow cytometry (Guava EasyCyte flow cytometer; Millipore, MA, USA).

### Cell cycle assay

The cells were cultured with compound 3K (1, 2.5, or 5 µM) or 0.1% DMSO (control), and the attached cells were harvested after 24 h and fixed with 70% cold EtOH at 4°C overnight. The cells were re-suspended in 0.5 ml phosphate buffered saline (PBS) containing 5 µl of staining solution (1 mg of PI in 1 ml of PBS) and RNase A (10 mg/ml), followed by incubation in the dark for 30 min at 37 °C. Next, the cells were analyzed using flow cytometry (Guava® EasyCyte flow cytometer).

### Acridine orange fluorescent staining

SK-OV-3 cells were cultured in confocal dishes, and after 24 h, they were treated with compound 3K (1, 2.5, or 5 µM) for an additional 24 h. The cells were stained with acridine orange (1 µg/ml) for 15 min and washed five times with PBS to remove the medium containing the dye. The cells were examined using a K1-fluo microscope (Nanoscope Systems, Daejeon, Korea) at 400× magnification.

### DAPI nuclear Staining

The nuclear morphology and presence of apoptotic bodies were visualized using DAPI nuclear staining. SK-OV-3 cells growing in a confocal dish were treated with compound 3K (1, 2.5, or 5 µM) for 24 h, and then the cells were fixed with acetone for 20 min. Next, the cells were washed five times with PBS, treated with (0.1 μg/ml DAPI in PBS for 5 min, and then re-washed with PBS. The nuclear morphology and apoptotic bodies in the cells were analyzed using fluorescence microscopy. Apoptotic cells were identified based on the characteristic changes including the presence of fragmented, condensed, and degraded nuclei.

### Immunocytochemistry for PKM2

SK-OV-3 cells were cultivated in confocal dishes and then treated with compound 3K (1, 2.5, or 5 µM). Next, the cells were fixed in acetone for 20 min, followed by removal with ice-cold PBS. Next, the cells were treated with 10% normal goat serum in PBS for 30 min before incubation with PKM2 antibody (1:500) at 4 °C overnight. The cells were washed five times with PBS and incubated with rhodamine conjugated secondary antibody for 30 min. Next, the cells were washed three times with PBS, stained with (0.1 μg/ml DAPI in PBS for 5 min, and washed again with PBS. Finally, the cells were examined under a K1-fluo microscope (Nanoscope Systems, Daejeon, Korea) at 400× magnification. Mean fluorescence intensity was analyzed using an image analyzer.

### Seahorse XF96 analysis of the extracellular acidification rate (ECAR)

A Seahorse XF96 analyzer (Agilent, Santa Clara, USA) was used to monitor the ECAR continuously. Two days prior to performing the experiment, the cells were grown in a XF96 well plate at a density of 2 × 10^3^ cells/well at 37 °C in 5% CO_2_. One day prior to the experiment, 200 µl of XF calibration buffer was added to each well of the XF cartridge, followed by incubation in an atmosphere of 0% CO_2_ at 37 °C overnight. Thirty minutes prior to the experiment, the cells were washed with PBS and 200 µl of XF assay medium was added to each well, followed by incubation for 30 min in an atmosphere of 0% CO_2_ at 37 °C. For the XF glycolysis stress analysis, 300 mg/L glutamine was added to the XF assay medium. After equilibration time, different compounds (10× final concentration) were added to the injection ports. The substrate consisted of 2 µM oligomycin, 10 mM glucose, and 50 mM 2-deoxyglucose. All the reagents for ECAR were purchased from Seahorse Bioscience.

### Quantification of lactate level in medium

Liquid chromatography (LC) system included a UV/Vis-151 detector (Gilson), an autosampler (Gilson-234), and a LC-321/322/350 pump (Gilson, France). Detection and quantification were performed using a Synergi Hydro-RP C18 column (250 × 4.6 mm, 4 μm, 80 Å; Phenomenex, USA). The flow rate was maintained at 0.8 ml/min for lactate. Isocratic mobile phases included 0.1% phosphoric acid in water for lactate. After extraction, the samples were transferred to a sample tube with acetonitrile containing thiamine (internal standard) and mixed thoroughly. The samples were centrifuged for 5 min at 1503×g. The supernatants were analyzed by performing HPLC (LC-321/322/350 pump) at 210 nm for lactate.

### IncuCyte ZOOM analysis

The effect of compound 3K on scratch-wound migration and cell confluency of ovarian cancer cells was examined using the IncuCyte ZOOM™ system (Essen Bioscience, MI, USA) that quantitatively detects live cells in real time. The cells were seeded and treated with compound 3K (1, 2.5, or 5 µM) for 48 h. To monitor cell confluency, the cells were plated in 96-well plates at a density of 3×10^3^ cells/well and incubated for 24 h. The cells were treated with compound 3K (1, 2.5, or 5 µM) and images were acquired every 6 h. All the photographs were analyzed, and confluency was calculated using the IncuCyte ZOOM™ software. The cells were seeded onto 96-well plates (IncuCyte Image-Lock Plates, Essen Bioscience) at a density of 1×10^4^ cells/well, 5% CO_2_, and 37 °C for performing the wound-healing assay. At 100% confluency, a WoundMaker (Essen Bioscience) was used to create a scratch-wound, and the cells were treated with compound 3K (1, 2.5, or 5 µM). The images were acquired every 6 h, and the relative wound healing density was calculated using the IncuCyte software. Each experiment was performed in at least six wells.

### *In vivo* tumor xenograft study

Female BALB/c nude mice (4-week-old) were obtained from Central Lab Animal Inc. (Hamamatsu, Japan). They were housed in controlled temperature (22 ± 2 °C) conditions and a 12 h environmental light/dark cycle. The experimental procedure was approved by the Sungkyunkwan University Institutional Animal Care and Use Committee (SKKUIACUC2019 -12-18-1). For the establishment of tumors, SK-OV-3 cells (1×10^7^) suspended in serum-free RPMI containing 50% Matrigel were injected subcutaneously in the right flank of the mice. The tumor volumes (V) were calculated every third day using a caliper and the following standard formula: V (mm^3^) = 0.52 (ab^2^) (a = length, b = width) 19. Once the tumor reached 200 cm^3^, the mice were randomly divided into two groups (n = 5 per group). According to previous studies 20, 21, compound 3K (5 mg/kg body weight) dissolved in 1 ml of solvent (DMSO:corn oil, 1:9) was administered orally every 2 days for 31 days. The control group was treated with an equal volume of the vehicle. The weights of the tumors were measured in each group. IHC analysis was conducted in the two groups, and the number of Ki-67 positive cells was analyzed using an image analyzer.

### Statistical analysis

All the data are expressed as the mean ± standard deviation (SD) of at least three independent experiments. The analysis of statistical significance was conducted using one-way analysis of variance (ANOVA) followed by Bonferroni's post-hoc comparisons test. Our analysis was conducted using Graph Pad Prism Software version 5.0 (GraphPad Software, CA, USA). A p value < 0.05 indicated statistical significance.

## Results

### PKM2 overexpression is correlated with severity of ovarian cancer

**Figure 1:**
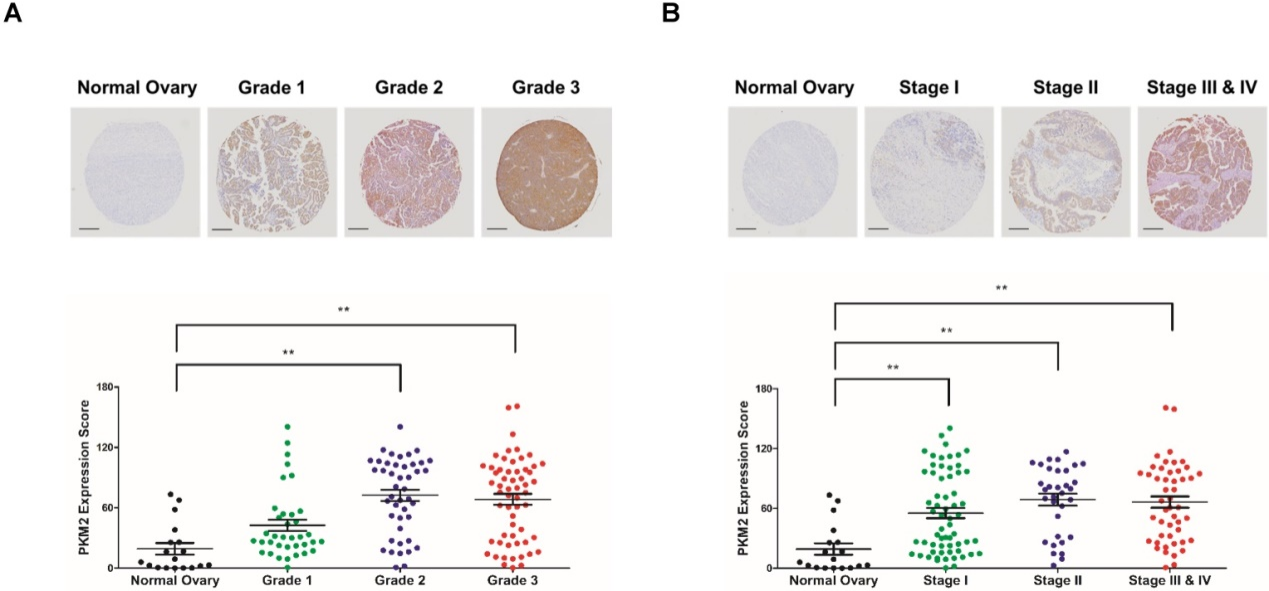
Expression pattern of PKM2 in tissue microarray. (A, B) Ovarian adenocarcinoma and normal ovarian tissue samples were analyzed for changes in PKM2 expression using immunohistochemistry. PKM2 immunostaining scores in the ovarian cancer and normal ovarian tissue samples associated with the four different tumor grades and stages. Scale bar, 200 µm. The values represent the mean ± SD. *p < 0.05 and **p < 0.01.

The expression of PKM2 was examined in 143 human ovarian adenocarcinoma tissue samples obtained from patients aged 22-75 years and compared with that in 18 normal ovarian tissue samples obtained from normal subjects aged 14-41 years. PKM2 expression had a positive correlation with the grade and stage of ovarian adenocarcinoma tissues (Figures 1A, B). These results indicate that a high expression of PKM2 is associated with poor survival of ovarian cancer patients.

### Cytotoxicity of compound 3K in SK-OV-3 cells

**Figure 2:**
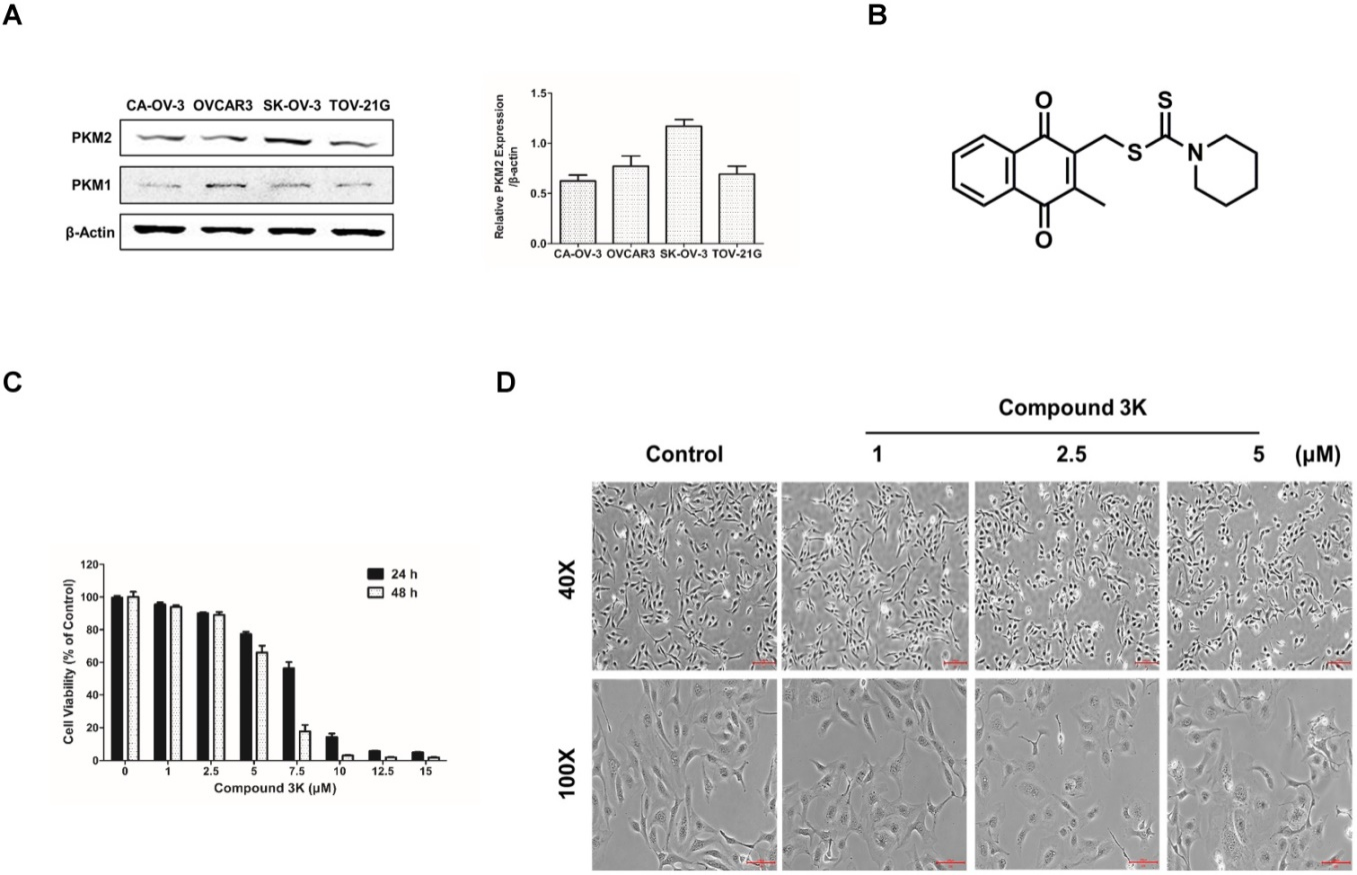
Protein expression level of PKM2 and PKM1 in ovarian adenocarcinoma cell lines and the effect of compound 3K on cell viability and cell morphology of SK-OV-3 cells. (A) Comparison of PKM2 and PKM1 expression level in four ovarian adenocarcinoma cell lines. β-actin served as a positive loading control. The band intensity of PKM2 was calculated and expressed as a ratio of β-actin expression in the graph. (B) The chemical structure of compound 3K. (C) Cells were treated with compound 3K at various concentrations (1-15 µM) for 24 and 48 h. Cell cytotoxicity was assessed using an 3-(4, 5-dimethylthiazol-2-yl)-2, 5-diphenyltetrazolium bromide (MTT) assay. (D) Light microscopic images show morphological changes in the cells after compound 3K (1, 2.5, or 5 µM) treatment for 24 h. Scale bar, 200 µm (40×), 100 µm (100×). The values represent the mean ± SD. *p < 0.05 and **p < 0.01.

A previous study reported a high expression of PKM2 in various cancer cells 22. We compared the basal expression of PKM2 and PKM1 in various ovarian cancer cell lines using western blot analysis and found that the PKM2 protein level was upregulated in the ovarian adenocarcinoma cell line, SK-OV-3, compared with the other ovarian cancer cell lines (Figure 2A). The chemical structure of compound 3K is presented in Figure 2B. The cytotoxicity of various concentrations of compound 3K in the SK-OV-3 cell line was evaluated using 3-(4,5-dimethylthiazol-2-yl)-2,5-diphenyl-tetrazolium bromide (MTT) assay. The IC_50_ value of compound 3K in the SK-OV-3 cells was assessed from a dose-response relationship curve. The cytotoxicity of compound 3K in the SK-OV-3 cells is shown in Figure 2C. Compound 3K exhibited potent cytotoxicity against SK-OV-3 cells in a time- and concentration-dependent manner, with IC_50_ values of 7.82 and 5.82 μM after 24 and 48 h of treatment, respectively. Noticeable morphological changes included expansion induced by compound 3K in the cells after 24 h of treatment (Figure 2D).

### Compound 3K affects the expression of PKM2 and not that of PKM1 in SK-OV-3 cells

**Figure 3:**
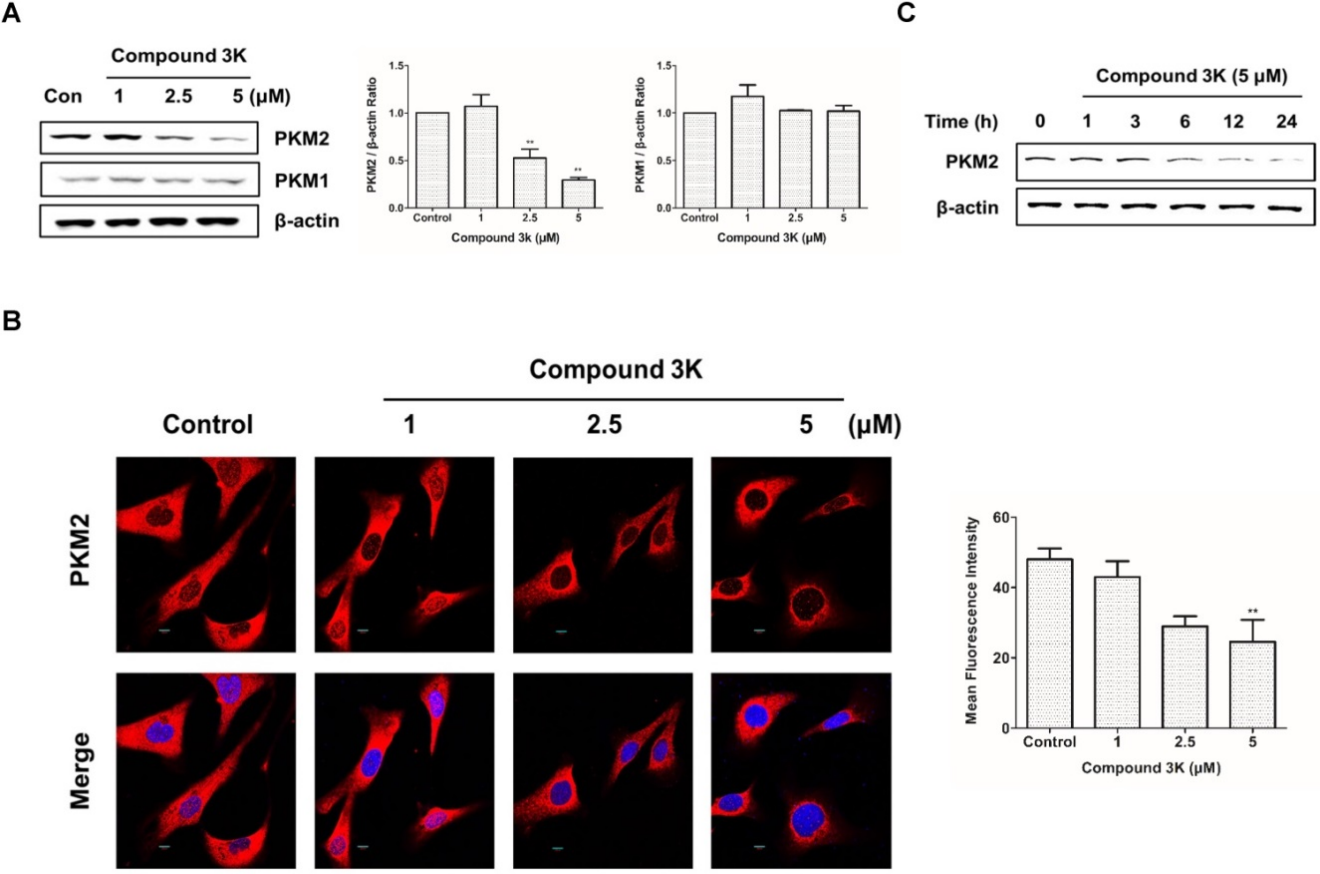
Effect of compound 3K on PKM2 and PKM1 expression. (A) Effect of compound 3K on the protein expression levels of PKM2 and PKM1. Cells were treated with compound 3K (1, 2.5, or 5 µM) for 24 h and expression level of the proteins was determined using western blotting. The band intensity of PKM2 and PKM1 were calculated and expressed as a ratio of β-actin expression in the graph. (B) Immunofluorescence of PKM2 labeled with an antibody to total PKM2 (Red). DAPI (blue) was used as a nuclear staining. (Magnification ×600). The mean fluorescence intensity of PKM2 was calculated and expressed in the graph. (C) Cells were treated with compound 3K (5 µM) for different time points, and then western blotting was conducted. The values represent the mean ± SD. *p < 0.05 and **p < 0.01.

Results of western blot analysis of whole-cell lysates showed a robust reduction in PKM2 protein expression in SK-OV-3 cells treated with compound 3K without any change in PKM1 protein expression (Figures 3A). Consistently, immunofluorescence analysis showed marked decrease in PKM2 protein levels in compound 3K-treated SK-OV-3 cells (Figure 3B). To determine the most effective treatment timepoint that completely inhibits PKM2 protein expression, cells were treated with 5 µM compound 3K for various timepoints ranging from 0 to 24 h. The maximum decrease in PKM2 expression was observed at 24 h (Figure 3C).

### Compound 3K inhibits proliferation and colony formation and regulates the cell cycle in SK-OV-3 cells

**Figure 4:**
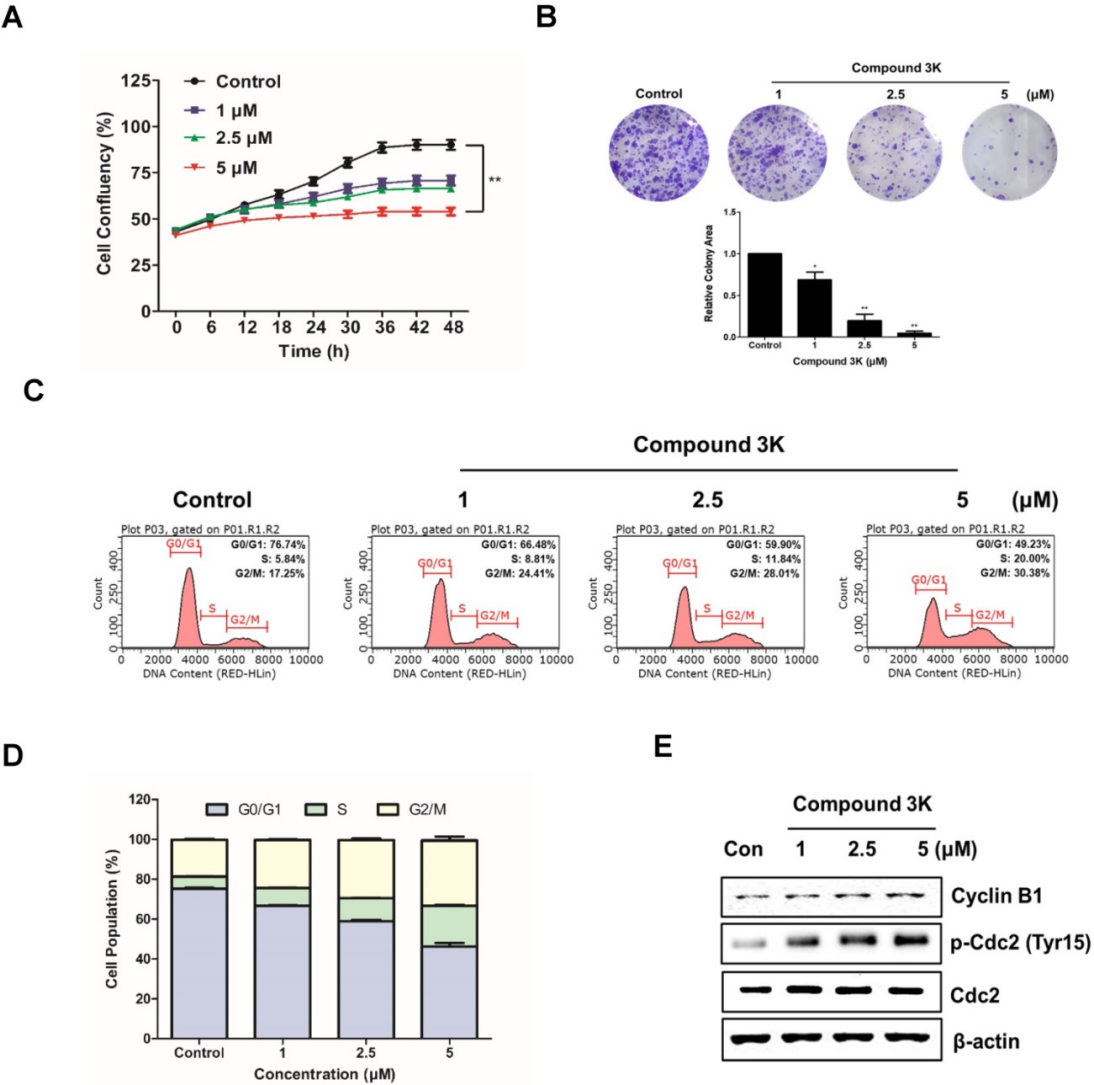
Compound 3K reduced colony formation and cell proliferation and induced the cell cycle arrest in SK-OV-3 cells. (A) Effect of compound 3K (1, 2.5, or 5 µM) on proliferation of SK-OV-3 cells following treatment for 48 h. Cell proliferation was checked with an IncuCyte software every 6 h. (B) Representative photographs and quantitative analysis of colony formation assay of SK-OV-3 cells treated with indicated compound 3K concentrations in 6-well plates. Colony areas were measured using image analyzer. (C) SK-OV-3 cells were treated with compound 3K (1, 2.5, or 5 µM) for 24 h. After treatment, the cells were stained with propidium iodide (PI) and analyzed using flow cytometry. (D) Bar graph indicating the different phases of cell cycle distributions of SK-OV-3 cells treated with 0.1% DMSO control or compound 3K. (E) Effect of compound 3K on the expression of cell cycle-related proteins. SK-OV-3 cells were treated for 24 h with compound 3K (1, 2.5, or 5 µM). The expression of cyclin B1, p-Cdc2, and Cdc2 were analyzed using western blot analysis. The values represent the mean ± SD. *p < 0.05 and **p < 0.01.

To evaluate the effect of compound 3K on the proliferation of an ovarian adenocarcinoma cell line, SK-OV-3 cells were treated with the indicated concentration (1, 2.5, or 5 μM) for 48 h. Cell confluency was calculated with the IncuCyte software, which quantitatively detects live cells every 6 h. Compound 3K noticeably reduced the proliferation ability of SK-OV-3 cells in a concentration-dependent manner with the maximum decrease in proliferation ability observed at 48 h (Figure 4A). To demonstrate the anticancer effects of compound 3K, a clonogenic assay was conducted. As shown in Figure 4B, colony formation of SK-OV-3 cells was markedly inhibited, following treatment with compound 3K. To identify the effect of compound 3K on the regulation of cell cycle, SK-OV-3 cells were treated with compound 3K (1, 2.5, or 5 µM) for 24 h; next, their DNA content was analyzed using flow cytometry. As shown in Figure 4C, compound 3K induced G2/M arrest of SK-OV-3 cells and simultaneously decreased the portion of cells in G1 phase in a concentration-dependent manner. The relative distribution of cells in the different phases of the cell cycle is displayed in Figure 4D. To identify the effect of compound 3K on the expression level of cell cycle-related proteins, western blotting was performed (Figure 4E). The expression levels of Cdc2 and cyclin B1 did not change significantly, whereas the expression level of p-Cdc2 increased in the compound 3K-treated SK-OV-3 cells. These results suggest that compound 3K inhibited the proliferation, colony formation, and cell cycle progression of SK-OV-3 cells.

### Compound 3K affects SK-OV-3 cell metastasis

**Figure 5:**
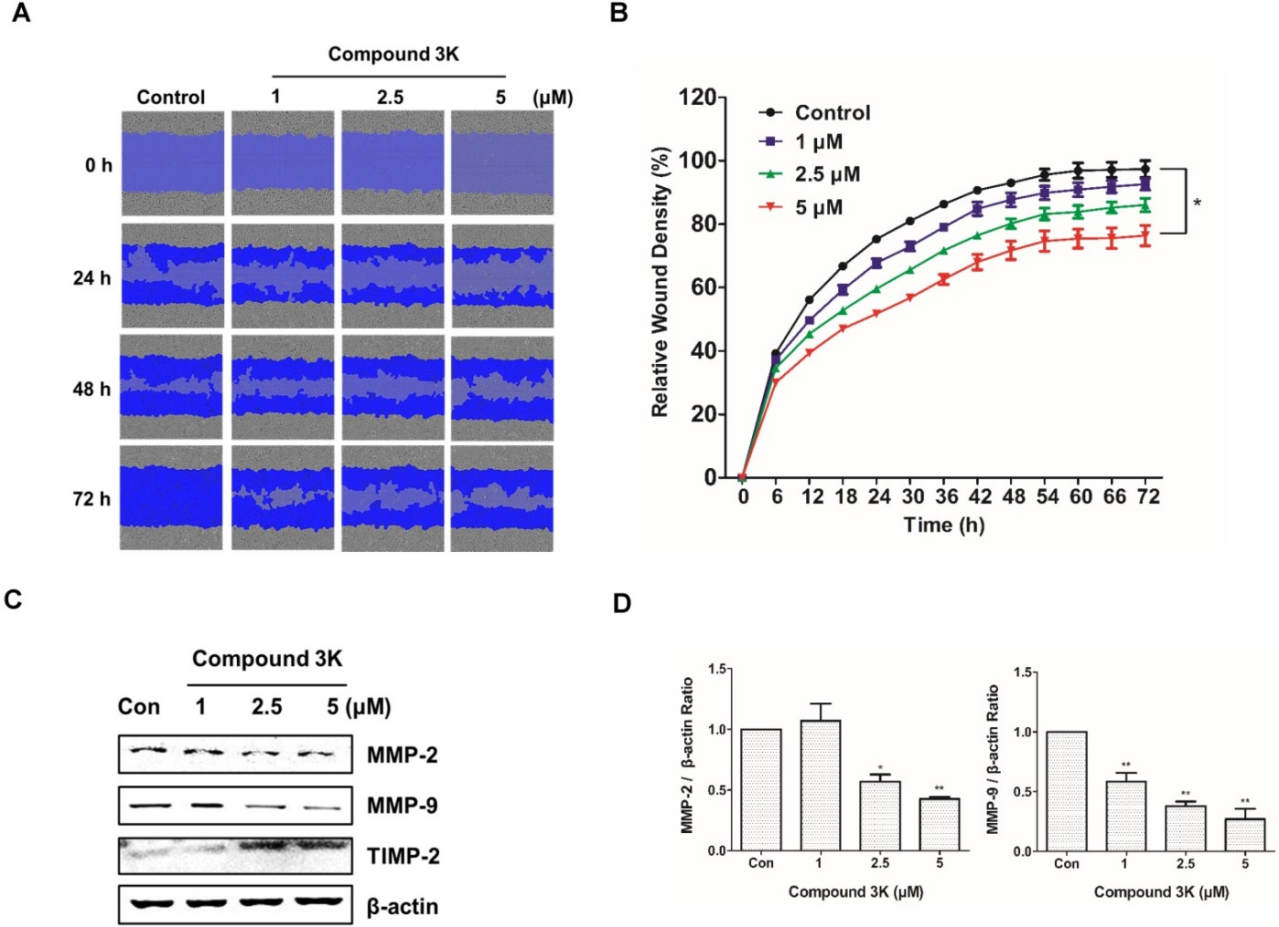
Compound 3K inhibits metastasis of SK-OV-3 cells. (A) The inhibitory effect of compound 3K on cell migration was evaluated using IncuCyte software. The blue area represents the wounded area. (B) The change in relative wound areas is shown. (C) Western blot analysis showing the protein levels of metastatic markers (MMP-2, MMP- 9, and TIMP-2) in SK-OV-3 treated with compound 3K (1, 2.5, or 5 µM). (D) Quantitative data of protein levels of the metastatic marker. The values represent the mean ± SD. *p < 0.05 and **p < 0.01.

Ovarian cancers are highly metastatic in the advanced stages 23. We investigated the effect of compound 3K on the migration of SK-OV-3 using a scratch-wound assay. The cells treated with compound 3K showed relatively lower wound healing ability than those in the control group (Figure 5A). The control and compound 3K-treated cells showed significant differences in relative wound healing at 72 h post-scratch (Figure 5B). These changes increased time-dependently. Additionally, the results of western blot analysis showed that compound 3K increased the protein expression of TIMP-2 and decreased that of matrix metalloproteinases protein (MMP) in a dose-dependent manner (Figures 5C, D). Thus, compound 3K inhibited the metastasis of SK-OV-3 cells.

### Compound 3K induces apoptotic and autophagic cell death in SK-OV-3 cells

**Figure 6:**
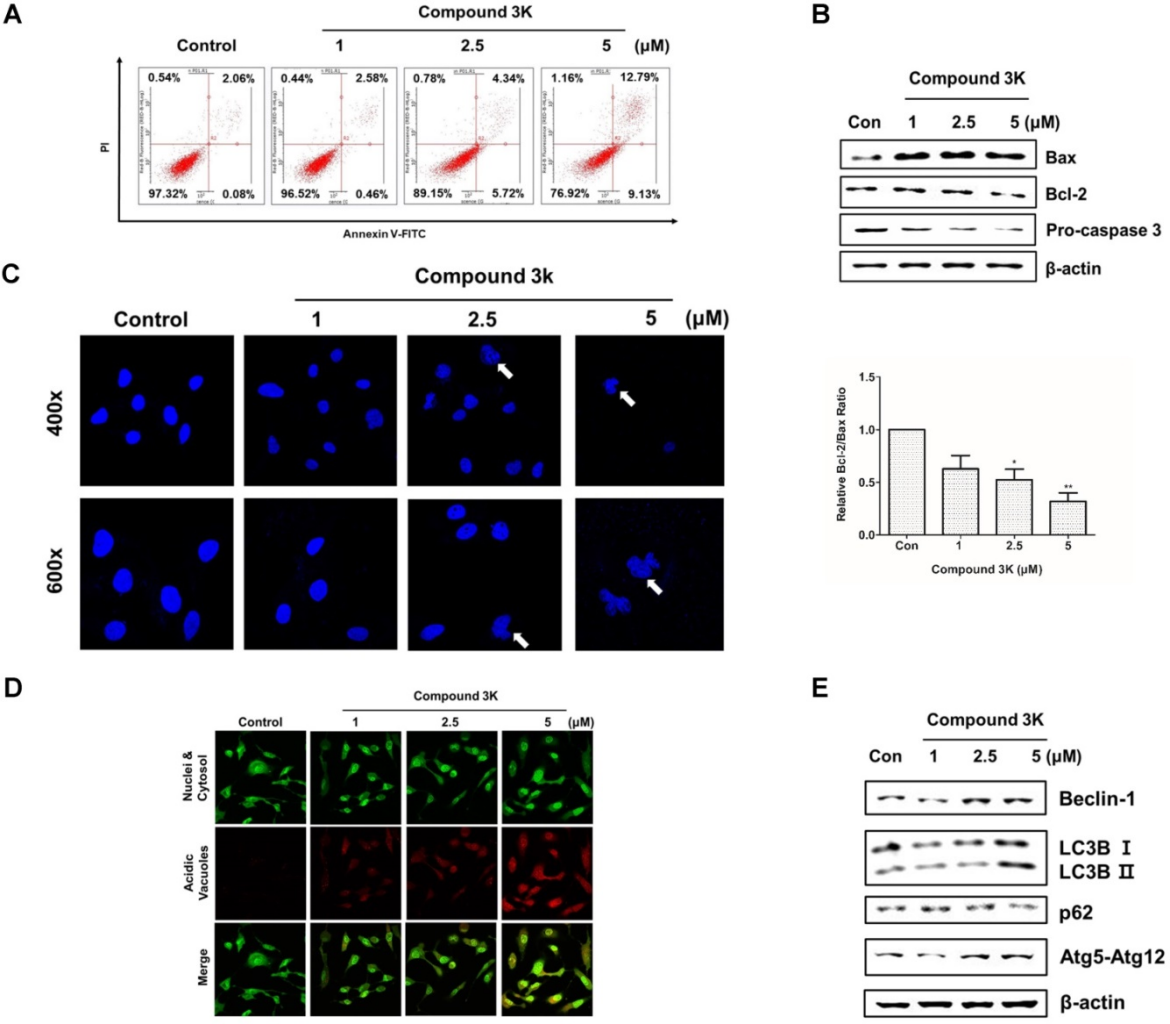
Apoptosis and autophagy induced by compound 3K in SK-OV-3 cells. (A) Cells were treated with vehicle control or compound 3K (1, 2.5, or 5 µM) for 24 h. After treatment, the cells were stained with PI and FITC-conjugated Annexin V for flow cytometric analysis. (B) Cells were treated with vehicle control or compound 3K (1, 2.5, or 5 µM) for 24 h and the expression level of pro-apoptotic proteins was analyzed by western blotting. The ratio of Bcl-2/Bax was calculated and expressed as a graph. (C) Effect of compound 3K on nuclear morphological changes determined by DAPI nuclear staining. Photographs were obtained using a confocal K1-fluo microscope (Nanoscope Systems, Daejeon, Korea) (Magnification ×400 and ×600). (D) Representative micrographs of acridine orange staining of SK-OV-3 cells following treatment with compound 3K (Magnification× 400). (E) Western blot analysis shows the protein levels of autophagy markers in SK-OV-3 treated with compound 3K (1, 2.5, or 5 µM) for 24 h. The values represent the mean ± SD. *p < 0.05 and **p < 0.01.

PKM2 regulation is associated with apoptosis and autophagy 17. Annexin V/PI assay, western blot analysis, and 4'-6-diamidino-2-phenylindole (DAPI) staining were performed to assess apoptotic cell death following compound 3K treatment. After 24 h, the proportion of the late-stage apoptotic cell population increased in the compound 3K-treated group, when compared with that in the control group (Figure 6A). Next, we assessed the expression level of apoptotic proteins using western blotting. Compound 3K increased the expression of Bax and decreased the expression of Bcl-2 and caspase 3 in SK-OV-3 cells (Figures 6B). DAPI nuclear staining was performed to examine the apoptotic cells using confocal microscopy. The compound 3K-treated group showed the presence of apoptotic nuclei (fragmented or condensed chromatin) (Figure 6C). To determine if autophagy was induced by compound 3K, acridine orange and western blot analysis were conducted. Autophagy activation was clearly observed by acridine orange staining of cells mediated by the accumulation of the lysotropic dye in acidic organelles in a pH-dependent manner 24. Compound 3K-treated cells showed autophagic vacuoles with higher red fluorescence intensity at 24 h (Figure 6D). The change of the soluble form of LC3-I to LC3-II, the autophagic vacuole-related form, is a major index of autophagosome formation. Compound 3K markedly increased the expression level of LC3-II. Moreover, the results showed a similar tendency in the expression of other relevant proteins including p62, Atg5-Atg12 complex, and Beclin-1 in the compound 3K-treated groups (Figure 6E).

### PKM2 regulates ovarian cancer cell growth through the AKT/AMPK/mTOR pathway

**Figure 7:**
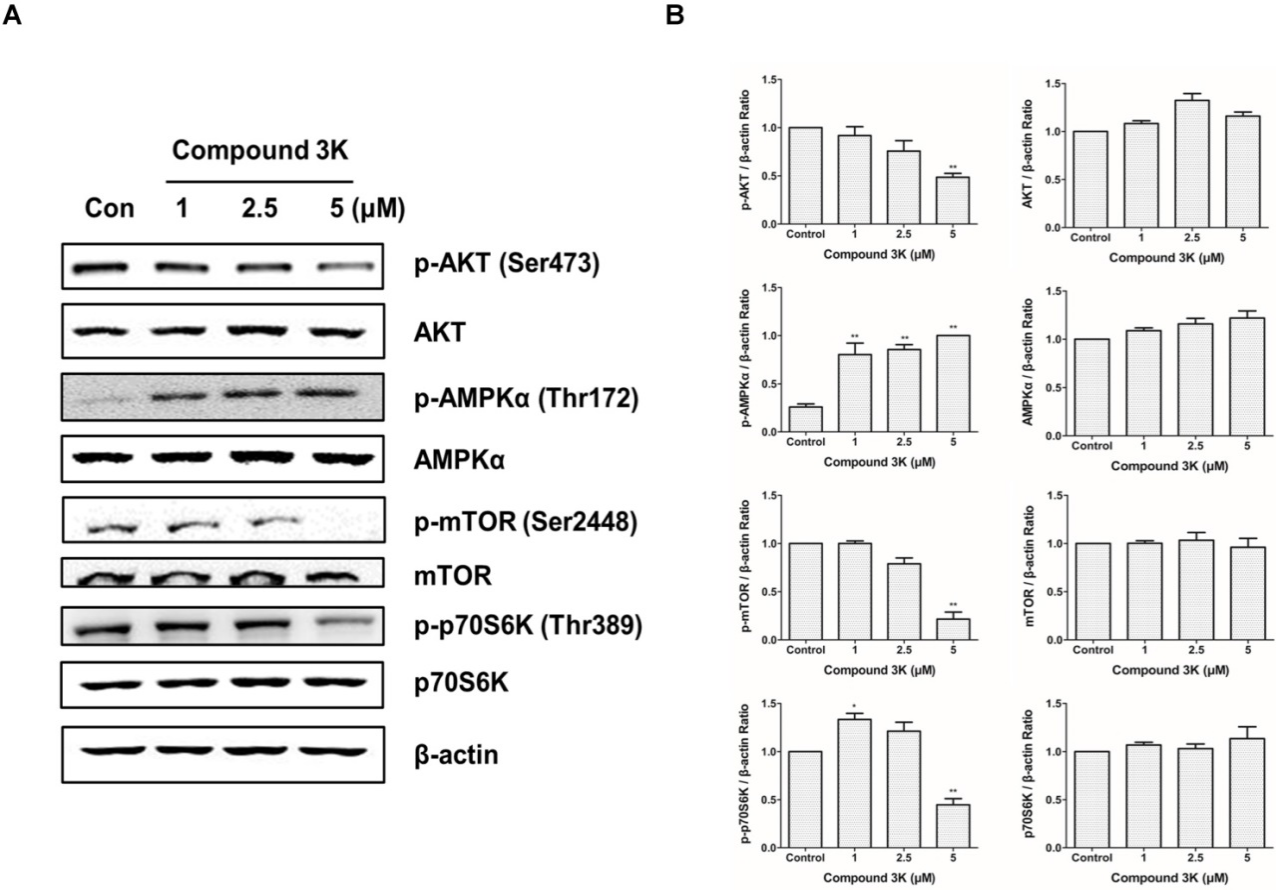
Changes in the expression of various proteins in signaling pathways following PKM2 inhibition by compound 3K for 24 h. (A) The effect of PKM2 inhibition on signaling pathways in SK-OV-3 cells. Cells were treated with compound 3K, and western blot analysis was conducted to determine the expression levels of various signaling pathway proteins. (B) The band intensity of Akt, p-Akt, AMPK, p-AMPK, mTOR, p-mTOR, p70S6K, and p-p70S6K were calculated and expressed in the graph as a ratio of β-actin. The values represent the mean ± SD. *p < 0.05 and **p < 0.01.

To determine the molecular mechanism through which PKM2 regulates the proliferation and growth of SK-OV-3 cells, we measured the expression levels of proteins associated with the PKM2-targeting molecular pathways in the compound 3K-treated SK-OV-3 cells using western blot analysis. Our results demonstrated that the PKM2/AKT/mTOR pathway was markedly inhibited in compound 3K-treated SK-OV-3 cells (Figures 7A, B). In addition, compound 3K significantly downregulated the expression of p-Akt, which led to the inhibition of mTOR phosphorylation and promotion of autophagy. The expression of Akt and mTOR was unchanged in the control group. Moreover, the inhibition of PKM2 increased the expression of p-AMPK, which in turn decreased mTOR phosphorylation. The induction of AMPK suppressed mTOR activity and negatively regulated protein synthesis; hence, we assessed the expression level of p-p70S6K, a main target of mTOR. Our data showed that p-p70S6K was inhibited in response to increased p-AMPK following PKM2 inhibition. These results indicate that the inhibition of PKM2 by compound 3K treatment resulted in a decrease in AKT phosphorylation and an increase in AMPK phosphorylation, causing decreased phosphorylation of mTOR and p70S6K.

### Compound 3K altered the glucose metabolism in SK-OV-3 cells

**Figure 8:**
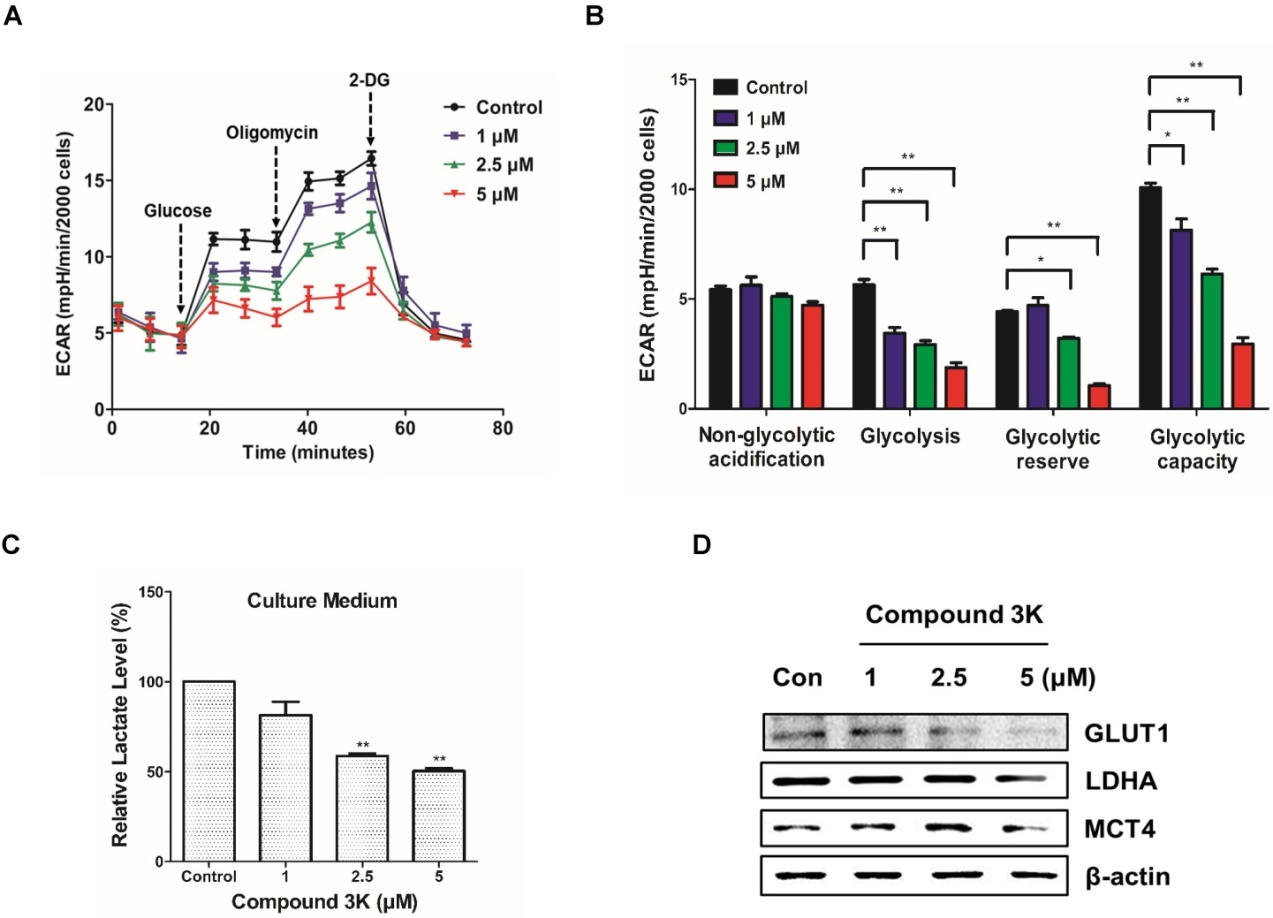
Compound 3K altered glycolytic function in SK-OV-3 cells. (A) The level of ECAR was measured using XF Glycolytic Stress Test kit. Following incubation for 24 h, compound 3K (1, 2.5, or 5 µM) was added to the plates followed by treatment with 15 mM glucose, 2 µM oligomycin, and 50 mM 2-DG subsequently. (B) Quantification of glycolytic function in SK-OV-3 cells following treatment. (C) Quantitative level of lactate in the culture media of compound 3K-treated cells. (D) Western blot analysis was performed to determine the protein expression levels of GLUT-1, LDHA, and MCT-4. The values represent the mean ± SD. *p < 0.05 and **p < 0.01.

To assess cellular bioenergetics in SK-OV-3, the Seahorse analyzer was used to identify changes in glycolytic function in the cells. The Seahorse analyzer measures ECAR, an indicator of glycolytic function, in cells during lactate production. As shown in Figure 8A, compound 3K decreased ECAR in a concentration-dependent manner. Glycolytic function can be estimated by evaluating various parameters such as glycolysis, glycolytic capacity, non-glycolytic acidification, and glycolytic reserve. There was a reduced glycolytic function in the compound 3K-treated groups compared with the control group (Figure 8B), indicating that compound 3K significantly induced glycolytic dysfunction. To identify the effect of compound 3K on the generation of cellular metabolites, we quantitatively analyzed lactate level in the culture media of the ovarian cancer cells using high performance liquid chromatography (HPLC). Compound 3K inhibited lactate production in a dose-dependent manner (Figure 8C). Furthermore, western blot analysis demonstrated that compound 3K reduced the expression levels of glucose transporter 1 (GLUT1), lactate dehydrogenase A (LDHA), and monocarboxylate transporter 4 (MCT4) considerably (Figure 8D), which might have caused decreased rates of glucose uptake, pyruvate conversion, and lactate efflux, respectively.

### Compound 3K inhibits cell growth in a SK-OV-3 tumor xenograft model

**Figure 9:**
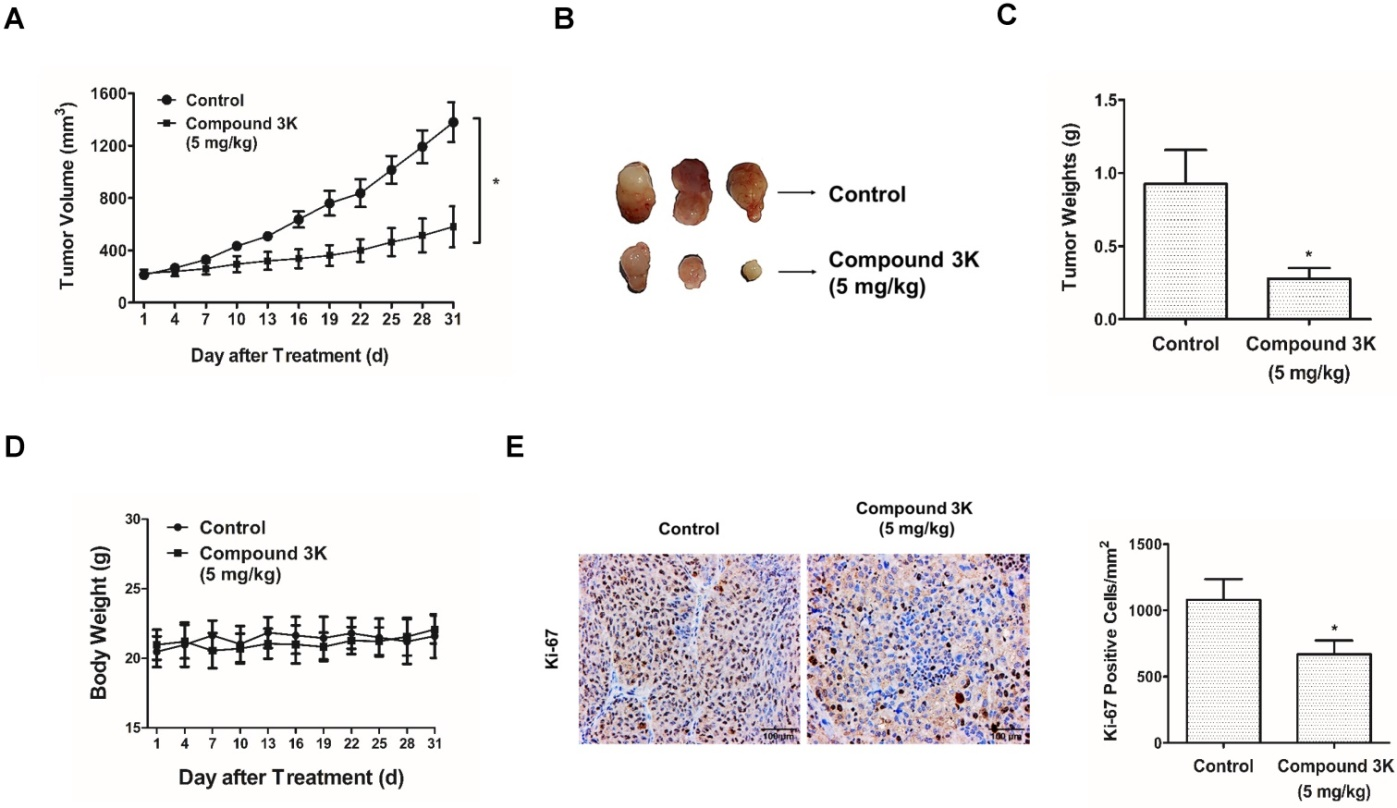
Compound 3K inhibited SK-OV-3 cell tumorigenesis *in vivo*. Compound 3K exhibited therapeutic effect on ovarian cancer in a tumor xenograft model. BALB/c nude mouse were divided into two groups randomly (n = 5 per group) and administered with vehicle or compound 3K (5 mg/kg) orally. Tumor size was calculated every three days for 31 days before the mice were sacrificed. (A) Changes in tumor volume. (B) Photographs of the tumors. (C) Comparison of tumor weight between compound 3K group and control group. (D) Changes of body weight. (E) Immunohistochemical analysis using an anti-Ki-67 antibody and its quantification (Magnification ×100). The values represent the mean ± SD. *p < 0.05 and **p < 0.01.

To identify the effect of compound 3K in a tumor xenograft model, SK-OV-3 cells (1×10^7^) were injected subcutaneously into BALB/c nude mice. The treatment began when the tumors were 200 cm^3^ and the mice were orally administered 5 mg/kg compound 3K for 31 days. Compound 3K treatment markedly decreased the tumor volume (Figures 9A, B) and tumor weight (Figure 9C), compared with the control group. No significant weight reduction was detected in the mouse treated with compound 3K (Figure 9D), suggesting that compound 3K did not cause any major organ toxicity. The proportion of Ki-67-positive cells is an important index of cell proliferation, and immunohistochemistry (IHC) analysis of the tumor tissue showed a high number of Ki-67-positive cells in the control group. However, the intensity of Ki-67 staining decreased in the tumor tissues from the compound 3K-treated mice (Figure 9E). All our results indicate that compound 3K inhibited SK-OV-3 cell growth *in vivo*.

## Discussion

Cancer cells rely heavily on PKM2 for their energy needs; it is an essential enzyme for the final rate-limiting step of the glycolytic pathway, and thus, it plays a critical role in cancer cell metabolism and growth 4. Clinically, PKM2 overexpression is associated with the tumorigenesis of various cancers and poor outcome for cancer patients 11, 25-27. In the current study, we observed a stronger PKM2 immunostaining in grades 2 and 3 and stages III and IV ovarian adenocarcinoma tissue samples, compared with that in normal ovarian samples, thus confirming that increased PKM2 expression is associated with ovarian tumorigenesis. The prognosis for patients with advanced ovarian cancer remains poor because of the ineffectiveness of chemotherapy or radiation therapy in ovarian cancers 2. Given that PKM2 overexpression is highly associated with advanced ovarian cancers, the development of novel anticancer drugs or therapeutics that can target PKM2 could be an important strategy to improve the survival rate of patients with PKM2-overexpressing ovarian cancer. To date, several PKM2 inhibitors, including shikonin, metformin, vitamin K, and temozolomide, have been identified 9. Our study focused on the effects of compound 3K, a specific PKM2 inhibitor, in ovarian cancer cells, and we mechanistically demonstrated that the effects of compound 3K were mediated by the targeting of metabolism for therapeutic benefit.

Although siRNA-PKM2 markedly inhibited cell proliferation and migration, induced apoptosis, and caused cell cycle arrest in SK-OV-3 cells overexpressing PKM2 14, the clinical application of siRNA therapy is limited. Therefore, to identify PKM2-targeted therapeutic drugs for potential clinical application, we evaluated the effects of compound 3K in SK-OV-3 cells, a specific inhibitor of PKM2 15. We investigated the mechanisms and *in vivo* toxicity of compound 3K in SK-OV-3 cells, a well-established human epithelial adenocarcinoma cell line model that is resistant to several cytotoxic drugs such as cis-platinum, diphtheria toxin, and adriamycin 18. We believe that our results will provide a basis for the application of PKM2 inhibitors in the treatment of advanced stages of ovarian cancer.

Specifically, we found that treatment with compound 3K markedly decreased the proliferation of SK-OV-3 cells in a dose and time-dependent manners, suggesting a relationship between the overexpression of PKM2 and ovarian cancer cell survival. A previous study reported that the knockdown of PKM2 in HeLa and SiHa cells at different cell cycle phases resulted in an increase in the percentage of cells in the G2/M phase. PKM2 knockdown increased DNA damage and G2/M cell cycle arrest following p53 activation, and thereby enhanced the radiosensitivity of the cells 28. In this study, treatment with compound 3K caused an arrest of the cells at the G2/M phase, which was mediated by an increase in the expression of p-Cdc2. Moreover, PKM2 plays an important role in the pathways associated with metastasis 6. SiRNA-mediated knockdown of PKM2 resulted in an upregulation of tumor suppressors, including Bad, caspase 7, and E-cadherin, whereas oncogenes, such as MMP-2, MMP-9, HIF1α, and VEGF, were downregulated in the SK-OV-3 cells 14. MMP-2 and MMP-9, which belong to the MMP family, are closely associated with the metastasis of tumor cells 29, 30.

Akt-mTOR signaling is a major negative regulatory pathway of autophagy 19, 31. Akt, the serine/threonine protein kinase, is associated with mTOR activity and positively regulates mTOR, which is a major protein in the regulation of autophagy 32. As a central control molecule, mTOR accepts signals from growth factors, nutrients, and many kinases including AMPK. The phosphorylation of AMPK induces a downstream signal that inhibits mTOR and activates autophagy, leading to the induction of catabolic processes 33. The phosphorylation of mTOR increases the expression of downstream proteins, such as p70S6K, to control various cellular processes. The phosphorylation of p70S6K is necessary for the induction of translation of proteins associated with proliferation and growth 34. Our data demonstrated that compound 3K regulated the Akt/AMPK/mTOR signaling pathway in SK-OV-3 cells. A previous study has shown that the knockdown of PKM2 activated AMPK signaling in response to the disturbed energy metabolism in H1299 cells. PKM2 knockdown increased AMPK phosphorylation at Thr172 and decreased p70S6K phosphorylation at Thr389. A549 cells that failed to induce AMPK signaling in response to PKM2 knockdown experienced apoptotic cell death but not autophagy. These results demonstrate the importance of AMPK signaling in cancer cell survival following energy disturbance caused by PKM2 inhibition 35.

Autophagy and apoptosis are crucial cellular processes that maintain cell homeostasis, and both processes are connected in many ways. Autophagy and apoptosis are coupled by the interaction of Bcl‑2 and Beclin‑1 36. The Bcl-2 family is a major regulator of apoptotic signaling associated with pro- and anti-apoptotic members. The post-translational phosphorylation of Akt, mTOR, and p70S6K regulates Bcl-2 activation 37. Beclin‑1, also known as the mammalian orthologue of yeast Atg6, is involved in the nucleation of the autophagosomal membrane. In cells, the Bcl‑2 family plays anti‑apoptotic roles 38. Beclin‑1 binds to the proteins of the Bcl‑2 family through a BH3 domain, and autophagy is activated by the release of Bcl‑2 from Beclin‑1 39. PKM2 knockdown influences the interaction between the members of Bcl‑2 family and Beclin‑1, which may be accompanied by a decreased expression level of Bcl‑2. A previous study showed that PKM2 knockdown activates autophagy and apoptotic signals in A549, an alveolar adenocarcinoma cell line 17. Our study also demonstrated that the PKM2 inhibitor, compound 3K, induced autophagy and apoptosis in SK-OV-3 cells.

**Figure 10:**
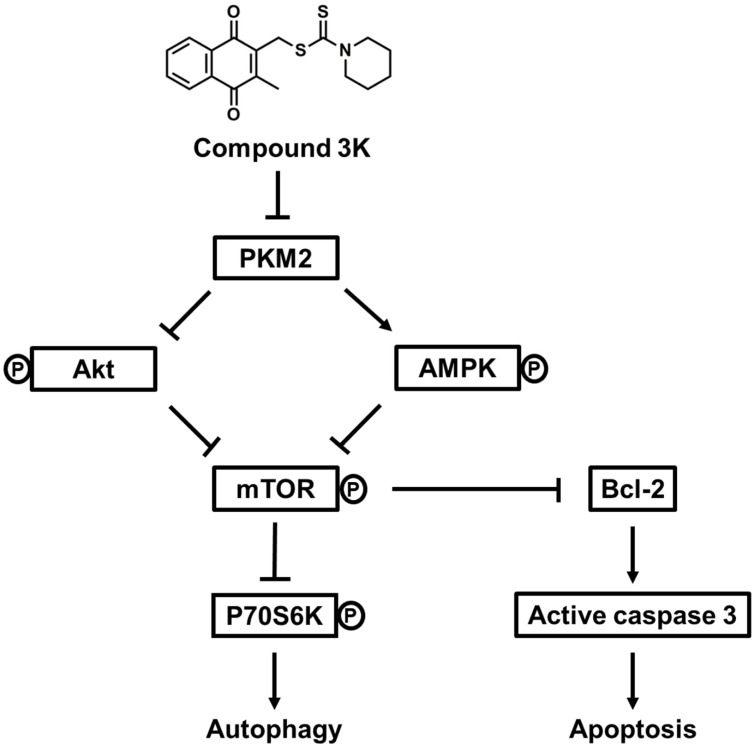
Schematic diagram illustrating potential pathways associated with cell death in compound 3K-treated SK-OV-3 cells. Compound 3K-induced cell death is associated with the Akt/AMPK/mTOR signaling pathway. Compound 3K induces phosphorylation of AMPK at Thr172 and reduces the phosphorylation of Akt. These molecular changes cause the suppression of mTOR signaling, thereby regulating autophagy and apoptosis.

In summary, our study indicated that compound 3K markedly inhibited glycolytic function and tumor growth in SK-OV-3 cells. Compound 3K markedly reduced the expression levels of GLUT1, LDHA, and MCT4, which may have inhibited glucose uptake, pyruvate conversion, and lactate efflux, respectively. Moreover, compound 3K treatment suppressed tumor growth and mass without significant body weight changes in the tumor xenograft mice. Collectively, our study demonstrated that compound 3K, a specific PKM2 inhibitor, inhibited SK-OV-3 cell growth and glycolytic function through autophagy and apoptosis mediated by the Akt/AMPK/mTOR signaling pathway (Figure 10).

## Figures and Tables

**Table 1:** Clinicopathological characteristics of the experimental microarray samples (143 ovarian adenocarcinoma tissue and 18 normal ovary tissue samples)

Variable	Patients (Female)	Tumor stage, n (%)			Histological grade, n (%)		
		I	II	III & IV	1	2	3
Cancer (%)	143 (100%)	62 (43.4%)	33 (23.1%)	48 (33.6%)	32 (24.6%)	40 (30.8%)	58 (44.6%)
Normal (%)	18 (100%)	-	-	-	-	-	-

## Abbreviations

PKM2: pyruvate kinase M2
PKM1: pyruvate kinase M1
MTT: 3-(4,5-dimethylthiazol-2-yl)-2,5-diphenyltetrazolium bromide
mTOR: mechanistic target of rapamycin
ATP: adenosine triphosphate
AKT1: AKT serine/threonine kinase 1
siPKM2: small interfering pyruvate kinase M2
PVDF: polyvinylidene difluoride
DMSO: dimethylsulfoxide
DAPI: 4',6-diamidino-2-phenylindole
LC: liquid chromatography
ANOVA: analysis of variance
IHC: immunohistochemistry
siRNA: small interfering ribonucleic acid
PBS: phosphate-buffered saline
GLUT1: glucose transporter 1
LDHA: lactate dehydrogenase A
MCT4: monocarboxylate transporter 4
RPMI: roswell park memorial institute medium
MMP: matrix metalloprotease
TIMP2: tissue inhibitor of metalloproteinases 2
